# Improved Exercise-Related Skeletal Muscle Oxygen Consumption Following Uptake of Endurance Training Measured Using Near-Infrared Spectroscopy

**DOI:** 10.3389/fphys.2017.01018

**Published:** 2017-12-12

**Authors:** Siana Jones, Andrew D'Silva, Anish Bhuva, Guy Lloyd, Charlotte Manisty, James C. Moon, Sanjay Sharma, Alun D. Hughes

**Affiliations:** ^1^Population Science and Experimental Medicine, Institute for Cardiovascular Science, University College London, London, United Kingdom; ^2^Cardiology Clinical and Academic Group, St. Georges University of London, London, United Kingdom; ^3^Barts Heart Centre, St Bartholomew's Hospital, London, United Kingdom

**Keywords:** endurance exercise, oxygen consumption, NIRS, skeletal muscle, VO_2_ kinetics

## Abstract

Skeletal muscle metabolic function is known to respond positively to exercise interventions. Developing non-invasive techniques that quantify metabolic adaptations and identifying interventions that impart successful response are ongoing challenges for research. Healthy non-athletic adults (18–35 years old) were enrolled in a study investigating physiological adaptations to a minimum of 16 weeks endurance training prior to undertaking their first marathon. Before beginning training, participants underwent measurements of skeletal muscle oxygen consumption using near-infrared spectroscopy (NIRS) at rest (resting muscle$\dot{\text{V}}$O_2_) and immediately following a maximal exercise test (post-exercise muscle$\dot{\text{V}}$O_2_). Exercise-related increase in muscle$\dot{\text{V}}$O_2_ (Δm$\dot{\text{V}}$O_2_) was derived from these measurements and cardio-pulmonary peak$\dot{\text{V}}$O_2_ measured by analysis of expired gases. All measurements were repeated within 3 weeks of participants completing following the marathon and marathon completion time recorded. Muscle$\dot{\text{V}}$O_2_ was positively correlated with cardio-pulmonary peak$\dot{\text{V}}$O_2_ (*r* = 0.63, *p* < 0.001). Muscle$\dot{\text{V}}$O_2_ increased at follow-up (48% increase; *p* = 0.004) despite no change in cardio-pulmonary peak$\dot{\text{V}}$O_2_ (0% change; *p* = 0.97). Faster marathon completion time correlated with higher cardio-pulmonary peak$\dot{\text{V}}$O_2_ (*r*_partial_ = −0.58, *p* = 0.002) but not muscle$\dot{\text{V}}$O_2_ (*r*_partial_ = 0.16, *p* = 0.44) after adjustment for age and sex [and adipose tissue thickness (ATT) for muscle$\dot{\text{V}}$O_2_ measurements]. Skeletal muscle metabolic adaptions occur following training and completion of a first-time marathon; these can be identified non-invasively using NIRS. Although the cardio-pulmonary system is limiting for running performance, skeletal muscle changes can be detected despite minimal improvement in cardio-pulmonary function.

## Introduction

Skeletal muscle metabolic adaptions with endurance exercise training are well-recognized (Gollnick et al., 1973; Holloszy and Booth, 1976). Recent studies implicate skeletal muscle metabolic dysregulation in disease and pre-disease states and a potential role for exercise in enhancing mitochondrial biogenesis has attracted recent interest (Lanza and Nair, 2009; Joseph et al., 2012). Skeletal muscle metabolic function is complex (Lanza and Nair, 2010) and studying muscle non-invasively is challenging. There is a pressing need to identify and apply sensitive, non-invasive measures of skeletal muscle metabolic function in order to investigate interventions that will deliver positive adaptations.

The effects of exercise training on the cardio-pulmonary system can be quantified using a cardio-pulmonary exercise test (CPET) to assess peak oxygen consumption (peak $\dot{\text{V}}$O_2_) *via* expired gas analysis. Multiple components contribute to peak $\dot{\text{V}}$O_2_ including: pulmonary diffusion, cardiovascular function (predominantly capacity to increase cardiac output, but also capacity for transport and exchange via the peripheral macro- and micro-circulation), the capacity for oxygen carrying in the blood, and cellular energy metabolism and mitochondrial function (Bassett and Howley, 2000). It is generally accepted that in humans, whole body peak $\dot{\text{V}}$O_2_, as measured by analysis of expired gases, is usually limited by the rate of oxygen delivery, and not by the rate of uptake/utilization in the muscle (Bassett and Howley, 2000). However, it remains unclear whether adaptation of one or other of these systems to a greater or lesser extent occurs. Previously, increased physical activity, but not necessarily heavy exercise training, has been show to enhance metabolic health independently of cardio-respiratory fitness (Laye et al., 2015).

Inclusion of a skeletal muscle assessments of oxygen consumption is uncommon within the context of a CPET. Vigelsø et al. (2014) have summarized evidence suggesting a direct positive correlation between improvements in peak $\dot{\text{V}}$O_2_ and muscle mitochondrial enzymatic activity. A limitation of these studies is that they require invasive muscle biopsy sampling and provide limited information about selected enzyme activity from a small tissue sample.

Near infrared spectroscopy (NIRS) is a non-invasive technique that can measure changes in oxygenated and deoxygenated hemoglobin (oxy-Hb and deoxy-Hb) in skeletal muscle up to a depth of ~1.5 cm (Grassi and Quaresima, 2016; Jones et al., 2016). Applying an arterial occlusion above the NIRS measurement site allows skeletal muscle oxygen consumption (muscle$\dot{\text{V}}$O_2_) to be measured (Hamaoka et al., 1985). This has been shown to have good reproducibility (van Beekvelt et al., 2001; Lacroix et al., 2012). Indices previously described in studies using NIRS have been based on response to exercise with, or without, arterial occlusions. Without arterial occlusions it is impossible to differentiate changes in oxygen consumption from changes in blood flow. It is not feasible to perform repeated arterial occlusions to estimate $\dot{\text{V}}$O_2_ throughout exercise, so a simple alternative is to assume that immediately post-exercise the $\dot{\text{V}}$O_2_ determined via arterial occlusion is equivalent to the $\dot{\text{V}}$O_2_ during the final stages of the exercising protocol (Southern et al., 2014). Short, transient arterial occlusions have also been applied in the post-exercise period to determine the kinetics of muscle$\dot{\text{V}}$O_2_ recovery (Ryan et al., 1985a; Motobe et al., 2004) these measurements have shown good reproducibility and agreement (Southern et al., 2014) with established ^31^P-MRI measurements of muscle oxidative capacity (Ryan et al., 1985a).

We aimed to (1) describe non-invasive local measurements of muscle$\dot{\text{V}}$O_2_ in the gastrocnemius at rest and immediately after peak exercise using NIRS. (2) compare muscle$\dot{\text{V}}$O_2_ with cardio-pulmonary peak $\dot{\text{V}}$O_2_ assessed simultaneously by CPET, and (3) investigate the effect of ~6 months of endurance training in healthy individuals preparing for a first marathon on muscle$\dot{\text{V}}$O_2_ and cardio-pulmonary peak $\dot{\text{V}}$O_2_, and how these related to first-time marathon running performance.

## Materials and methods

### Participants

Participants were eligible for inclusion in this analysis if they had been enrolled in an observational study investigating the effect of first time marathon running on cardiac remodeling: The Marathon Study. Inclusion criteria for The Marathon Study were: age 18–35 years old at recruitment, no past significant medical history, no previous marathon-running experience, and current participation in running for <2 h per week. This analysis is a sub-study of The Marathon Study. Appendix 1 (Supplemental Information) shows full details of flow of participants through this study and selection for this analysis.

All measurements were carried out before training at ~6 months prior to the marathon (pre-training) and were repeated within a 3 week window following completion of the Marathon (post-training). The study design is shown in the diagram in Appendix 2 (Supplemental Information).

All procedures were in accordance with the principles of the Helsinki declaration, all participants gave written informed consent and the study was approved by the London—Queen Square National Research Ethics Service (NRES) Committee-−15/LO/0086.

### Training

Participants were recommended to adhere to the “First-time finisher” training schedule (http://london-marathon.s3.amazonaws.com/vmlm2014/live/uploads/cms_page_media/1161/First-time-finisher-2017.pdf), aiming to run ~3 times per week for the 16 week period prior to the marathon. However, those eager to follow alternative, higher intensity, training plans were not discouraged. Some participants wished to start their training earlier than 16 weeks prior to the marathon and so we elected to conduct pre-training testing immediately following the release of the results from the ballot entry system which was 6 months prior to the marathon. Therefore, the minimum training period for all participants was 16 weeks but some participants may have trained for up to 26 weeks before the repeat assessment.

### Anthropometric measurements

Participants were not asked to fast prior to either pre-training or post-training study visit but were asked to abstain from heavy exercise in the 24 h prior. We did not control for hydration during the study visits, however, participants were offered water throughout their visit but were asked not to have caffeinated drinks.

Height was measured barefoot using a standard stadiometer. Weight, body fat mass, and body fat percentage were measured using digital bio-impedance scales (BC-418, Tanita, USA) which have previously been shown to have good test-retest reproducibility (Kelly and Metcalfe, 2012). Body fat was included in this analysis because of the previously described substantial effect of adipose tissue thickness (ATT) on the NIRS signal (van Beekvelt et al., 2001).

### Cardio-pulmonary exercise test

The CPET was carried out on a semi-supine ergometer (Ergoselect1200, Ergoline, Germany) using an incremental protocol standardized by bodyweight and gender. A semi-supine ergometer was used to allow concurrent echocardiography to be performed. Expired gases were analyzed throughout using a metabolic cart system (Quark CPET, COSMED, Italy). All participants were required to achieve a respiratory exchange ratio (RER) value above 1.1 during the CPT, however, the test was not terminated until the participant reported exhaustion or could no longer maintain the cycling cadence (~60–70 rpm).

Peak $\dot{\text{V}}$O_2_ was determined as the rolling 30 s average value between the final 15 s of exercise and first 15 s of recovery. Maximal predicted $\dot{\text{V}}$O_2_ was calculated according to the equation by Wasserman et al. (1999) and the percentage of this predicted value reached during the exercise test was calculated. Resting heart rate was measured using a 12-lead ECG conducted in the semi-recumbent position, 45° to the horizontal, following a 2–5 min resting period. Peak heart rate was the highest rolling 60 s average value during the final 30 s of exercise.

### Skeletal muscle measurements

The gastrocnemius was selected for investigation because it is recruited during running and cycling (Zehr et al., 2007), it is therefore likely to undergo adaptations with training. NIRS measurements from this site have been shown to be reproducible (Southern et al., 2014) and since the calf is covered by less adipose tissue than the thigh or larger locomotive muscles (Bielemann et al., 2016) the influence of ATT on NIRS measurements is less (van Beekvelt et al., 2001). Prior to attaching the NIRS device, ATT was measured at the site of NIRS measurement using B-mode ultrasound (Vivid I, GE healthcare) equipped with a 12L-RS linear array transducer; three measurements were averaged.

A continuous wave NIRS device (Portamon, Artinis Medical Systems, Netherlands) was used to measure changes in oxy-Hb and deoxy-Hb from the lateral gastrocnemius. Position and orientation of the device was standardized between individuals. The device was attached using tape and covered completely using a neoprene sleeve. Measurements were acquired at a frequency of 10 Hz throughout the protocol.

For NIRS measurements the participant was seated in a semi-upright position with their gastrocnemius relaxed. Arterial occlusions were performed at rest and immediately following the exercise test using a rapid inflation cuff (Hokanson, SC10D/E20, PMS Instruments, UK) placed on the thigh directly above the patellofemoral articulation. The cuff was inflated to a pressure of 250 mmHg and inflation or deflation was complete in 0.3 s. At rest the cuff was inflated for 30 s, post-exercise the occlusion duration was 5–8 s.

### NIRS post-processing

Analysis of NIRS data was conducted using custom written programs in MATLAB R2014a (MathWorks Inc.). Signals were rejected if visual inspection showed movement artifact or if there was evidence that complete arterial occlusion had not be achieved. Incomplete arterial occlusion was judged on the absence of pulsatility in the oxy-Hb signal and the direction of the oxy-Hb and deoxy-Hb signals; under complete arterial occlusion these are expected to move in opposite directions.

Muscle$\dot{\text{V}}$O_2_ was estimated by fitting the slope of the difference between oxy-Hb and deoxy-Hb signal during each occlusion (Ryan et al., 1985b). More negative values represent higher muscle oxygen consumption; therefore, in order to align our values with the cardio-pulmonary peak $\dot{\text{V}}$O_2_, we inverted the values to provide a positive indices of muscle oxygen consumption. Exercise-related increases in muscle$\dot{\text{V}}$O_2_ (Δmus$\dot{\text{V}}$O_2_) were calculated as the absolute difference between resting muscle$\dot{\text{V}}$O_2_ and muscle$\dot{\text{V}}$O_2_ measured immediately post-exercise.

Therefore, three indices of muscle function were calculated for each participant that represent oxygen consumption at different times; resting muscle$\dot{\text{V}}$O_2_, post-exercise muscle$\dot{\text{V}}$O_2_, and Δmus$\dot{\text{V}}$O_2._ The units of these measurements are micoMolars of the hemoglobin difference signal per second (μM-Hb_diff_/s).

### Statistical analysis

Categorical data (i.e., gender) are presented as number (percentage) of participants. Continuous data were examined for normality and participant characteristics are presented as mean ± standard deviation or median (interquartile range) if skewed. Results are presented as means (95% confidence intervals). Comparison of means was done using a paired Student's *t*-test and comparison of skewed data using a Wilcoxon signed-rank test. Pearson's correlation coefficient (*r*) was calculated to assess correlations. Partial correlation coefficients (*r*_partial_) were calculated for relationships between marathon completion time and peak$\dot{\text{V}}$O_2_ or muscle$\dot{\text{V}}$O_2_ after adjustment for age and gender, or age, gender, and log(ATT) as appropriate. To check for any possible gender differences in associations we included an interaction term in multivariable models and also undertook analyses stratified by gender. Gender stratified data are presented in the Appendix 3 (Supplemental Information); as no significant gender interactions were observed, we performed the primary analysis without stratification and present results as partial correlation coefficients with adjustment for gender but without interaction by gender. Statistical significance was assigned if *P* < 0.05.

## Results

### Participants

Twenty-seven participants provided complete data for analysis, participant characteristics are given in Table 1. The mean duration from completion of the marathon to the post-training visit was 15.8 ± 4.6 days.

**Table 1:** Participant characteristics at pre-training.

**Mean ±*SD***	**Baseline (*n* = 27)**
Gender male (%)	17 (63)
Age (years)	29.4 ± 3.5
Height (cm)	176.7 ± 10.4
Weight (kg)	72 ± 13
Body fat mass (kg)	15 ± 5.5
ATT_gastroc_ (cm)	0.60 ± 0.22

*Data are mean ± SD*.

### Muscle $\dot{\text{V}}$O_2_ and cardio-pulmonary peak$\dot{\text{V}}$O_2_

**Figure 1:**
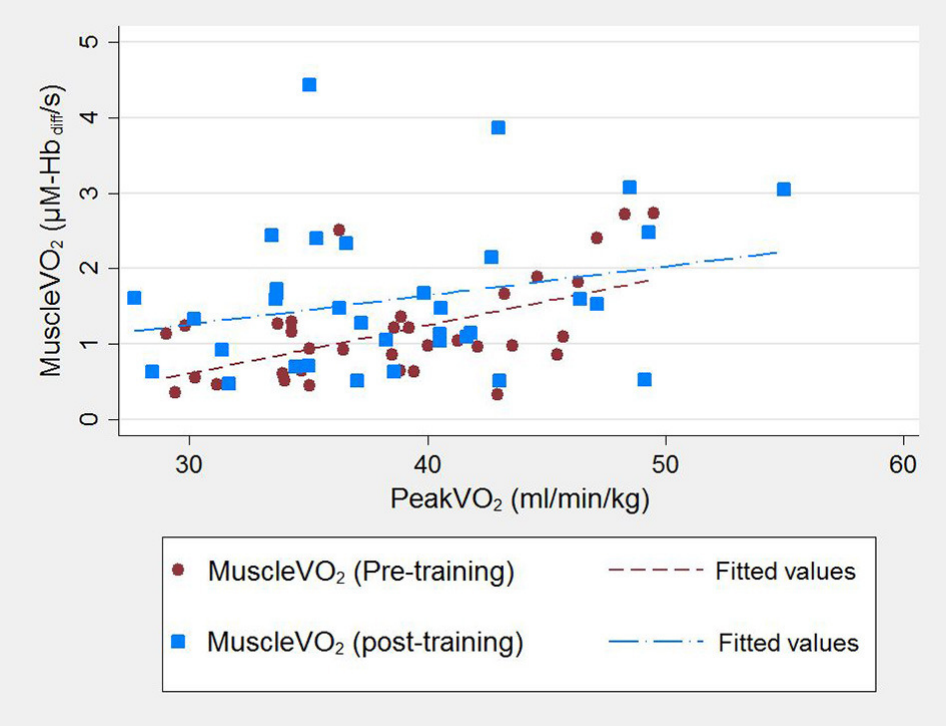
Correlationbetween cardio-pulmonary peak$\dot{\text{V}}$O_2_ and post-exercise muscle$\dot{\text{V}}$O_2_ for 27 participants assessed pre-training and post-training. Participant data pre-training are represented by red circles and a red line of best fit and post-training by blue squares and a blue line of best fit.

Pre-training higher post-exercise muscle$\dot{\text{V}}$O_2_ correlated with higher cardio-pulmonary peak$\dot{\text{V}}$O_2_ (Pearson's *r* = 0.64, *p* < 0.001; Figure 1). This correlation was marginally attenuated by adjustment for logATT (*r*_partial_ = 0.57, *p* = 0.002). Post-training a positive correlation was also seen but it was less strong [Pearson's *r* = 0.27, *p* = 0.17 (Figure 1); *r*_partial_ = 0.16, *p* = 0.4 after adjustment for logATT].

### Effect of endurance training

**Figure 2:**
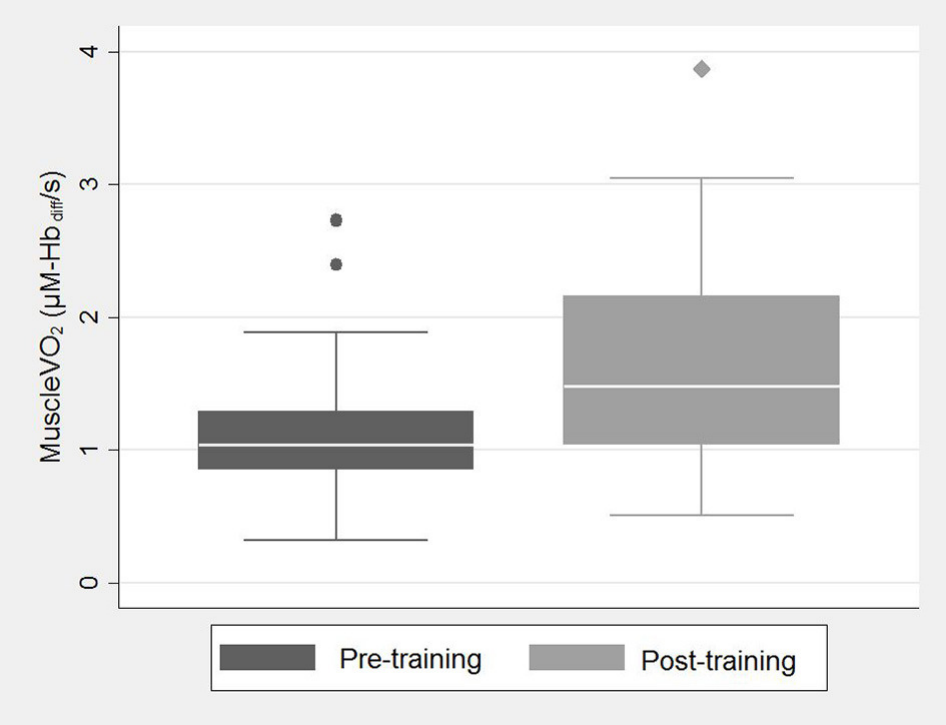
Box and whisker plot showing post-exercise muscle$\dot{\text{V}}$O_2_ pre-training (dark gray) and post-training (light gray). A box and whisker plot is used to describe this data because of its skewed distribution. The bottom and top of the box represent the first and third quartiles, the band inside the box is the median and the whiskers represent the lower and upper adjacent values within 1.5*IQR of the lower and upper quartile, respectively. Medians were compared using a Wilcoxon signed-rank test; follow-up values were significantly higher than baseline.

**Figure 3:**
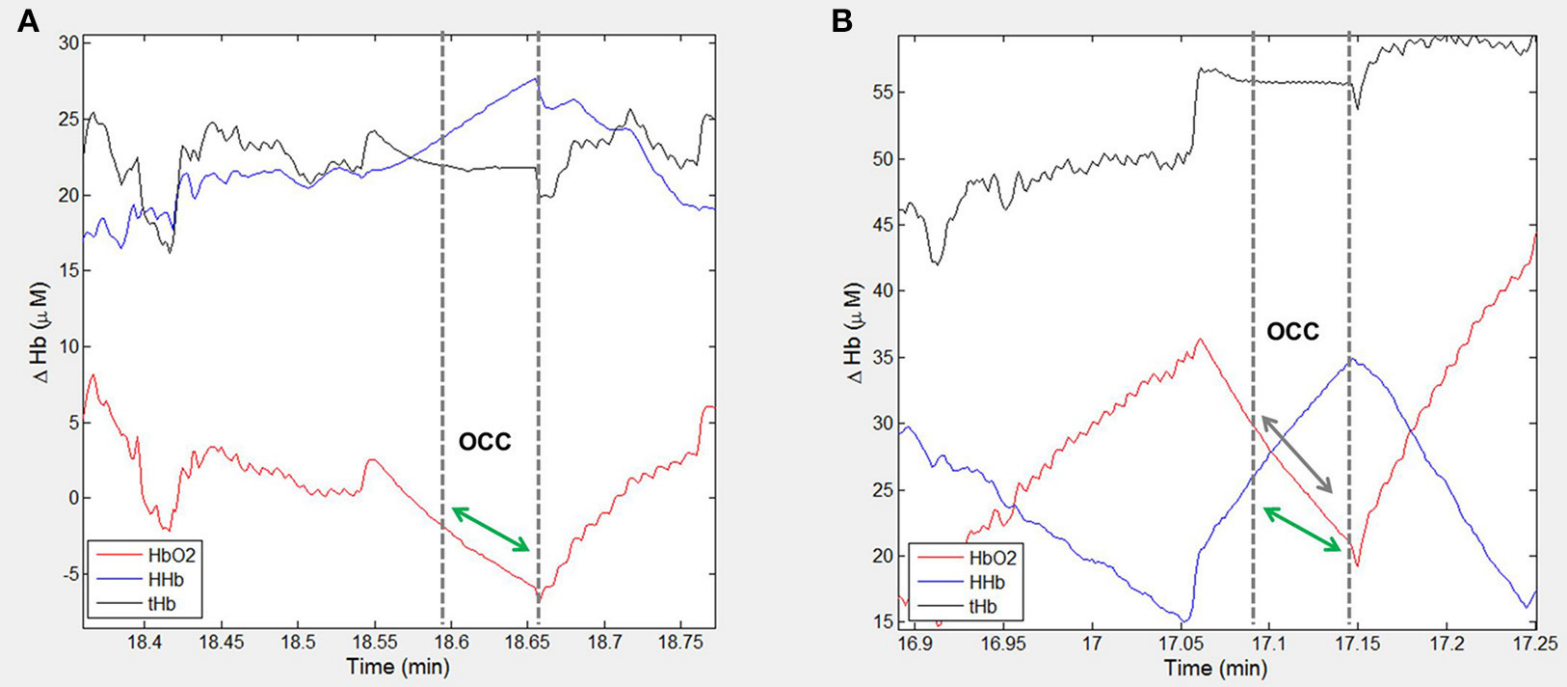
Example participant data showing the NIRS signal during arterial occlusions (OCC) performed immediately post-exercise. Changes in oxygenated hemoglobin (HbO_2_), deoxygenated hemoglobin (HHb) and total hemoglobin (tHb) are shown for one participant at **(A)** pre-training visit and **(B)** post-training visit. The dashed lines indicate the measurement area and green arrow indicates the slope of decline in HbO_2_ which represents rate of oxygen consumption in the muscle. The green arrow is shown at both time-points to highlight the change with training.

There was a small reduction in resting heart rate measured pre-training compared to post-training [mean difference = 1.4 (95% CI: −4.5, 7.2) bpm, *p* = 0.63; Table 2]. Cardio-pulmonary peak$\dot{\text{V}}$O_2_ did not change with training [mean difference = −0.0 (95% CI: −2.1, 2.0) ml.min.kg, *p* > 0.9; Table 2], but post-exercise muscle$\dot{\text{V}}$O_2_ was greater fpost-training compared to measurements pre-training [median value(IQR); 1.04(0.85–1.29) vs. 1.48(1.04–2.16), *p* = 0.004; Figure 2, Table 2]. Figure 3 shows example participant data of the NIRS signals during arterial occlusions (OCC) performed immediately post-exercise at the pre-training visit and post-training visits (Figures 3A,B, respectively). The dashed lines indicate the measurement area and green arrow indicates the slope of decline in HbO_2_ which represents rate of oxygen consumption in the muscle. The green arrow is shown at both time-points to highlight the change with training. Δmus$\dot{\text{V}}$O_2_ was also greater post-training [mean difference = −0.79 (95% CI:−1.1, −0.46) μM-Hb_diff_/s; *p* < 0.001]. There was a small reduction in resting muscle $\dot{\text{V}}$O_2_ [median value(IQR); 0.25(0.19–0.32) vs. 0.21(0.16–0.32), *p* = 0.35; Table 2].

**Table 2:** Physiological response to exercise for all participants.

**Mean ±*SD* or Median (IQR)**	**Pre-training**	**Post-training**	***P*-value**
Weight (kg)	72.0 ± 13.0	72.0 ± 12.0	0.98
BMI	22.9 ± 2.9	23.0 ± 2.7	0.84
Body fat mass (kg)	15.0 ± 5.5	14.4 ± 6.2	0.30
Resting HR (bpm)	66 ± 13	64 ± 14	0.63
Peak HR (bpm)	167 (161–176)	165 (157–171)	0.29
Peak VO_2_ (ml/min/kg)	39.2 ± 5.7	39.2 ± 6.7	0.97
Perc. pred. VO_2_ (%)	94.1 ± 13.4	94.1 ± 13.5	0.98
Resting muscle VO_2_ (μM-Hb_diff_/s)	0.25 (0.19–0.32)	0.21 (0.16–0.32)	0.35
Post-Ex muscle VO_2_ (μM-Hb_diff_/s)	1.04 (0.85–1.29)	1.48 (1.04–2.16)	0.004
ΔmusVO_2_ (μM-Hb_diff_/s)	0.88 ± 0.56	1.45 ± 0.94	<0.001

*Data are mean ± SD for normally distributed data and median(IQR) for non-normally distributed data. Comparison of means was done using a paired Student's t-test and comparison of skewed data using a Wilcoxon signed-rank test*.

### Marathon performance

The mean marathon completion time was 4.6 ± 0.81 h. This was longer for women vs. men (4.9 ± 1.0 vs. 4.3 ± 0.6 h, *p* = 0.07). Based on weekly mileage data and marathon completion times from 27,000 runners over a 16 week training period (Reese et al., 2014) the average times achieved by participants in this study are consistent with a training schedule of between 6 and 13 miles/per week.

Higher cardio-pulmonary peak$\dot{\text{V}}$O_2_ values post-training were associated with faster marathon completion times after adjustment for gender and age (*r*_partial_ = −0.58, *p* = 0.002). Greater increases in peak$\dot{\text{V}}$O_2_ from the pre-training value also predicted shorter marathon completion times (*r*_partial_ = −0.53, *p* = 0.007). Table 3 shows individual participant peak$\dot{\text{V}}$O_2_ measured pre-training and post-training and the corresponding marathon completion time. There was no convincing relationship between post-exercise muscle$\dot{\text{V}}$O_2_ measured post-training and marathon completion time (*r*_partial_ = 0.31, *p* = 0.14) or Δmus$\dot{\text{V}}$O_2_ and marathon completion time (*r*_partial_ = 0.31, *p* = 0.14).

**Table 3:** Peak VO_2_ (ml/min/kg) is described for individual participants at the pre-training measurement visit and post-training.

**Peak VO**_**2**_ **(ml/min/kg)**		**Marathon time (h)**
**Pre-training**	**Post-training**	
39.22	39.87	3.43
36.46	47.13	3.48
44.62	46.40	3.52
42.11	43.04	3.71
42.95	49.14	3.82
38.59	41.81	3.98
33.70	33.64	4.04
40.00	40.54	4.14
35.05	36.60	4.14
47.10	49.28	4.15
49.51	54.96	4.27
43.56	37.21	4.28
38.91	42.70	4.31
34.30	38.25	4.41
45.70	33.67	4.48
34.73	34.45	4.70
34.29	35.33	4.73
43.22	41.63	4.73
33.90	37.04	4.79
38.52	40.51	4.85
48.29	33.47	5.03
29.43	31.39	5.04
41.28	40.55	5.12
31.17	30.20	5.22
45.45	42.99	5.54
35.06	28.45	6.37
29.82	27.69	6.86

*Marathon completion time is also given*.

## Discussion

This study measured rest and post-exercise muscle$\dot{\text{V}}$O_2_ using NIRS simultaneously with an assessment of cardio-pulmonary peak$\dot{\text{V}}$O_2_ via CPET in a sample of young, healthy men and women before and after 4–6 months of endurance training in preparation for a first-time marathon. Compared with pre-training, post-training was associated with an increase in post-exercise muscle$\dot{\text{V}}$O_2_ and Δmus$\dot{\text{V}}$O_2_, despite no detectable change in cardio-pulmonary peak$\dot{\text{V}}$O_2_.

The magnitude of the increase in muscle$\dot{\text{V}}$O_2_ was similar to that previously reported in healthy volunteers using NIRS following bicycle training (vastus lateralis; Sako, 2010) or training of arm muscles (Ryan et al., 2013), or using succinate dehydrogenase (Green et al., 1985) or citrate synthase (Burgomaster et al., 2008) as indicators of muscle oxidative capacity. However, one study failed to observe an increase in maximum muscle oxygen consumption in finger flexor muscles following 6 weeks of training with a dynamic handgrip exercise (Fujioka et al., 2012). Our findings suggest that, in these young, healthy individuals, a comparatively low-level of endurance training, while not sufficient to noticeably improve cardio-pulmonary function, still led to positive skeletal muscle adaptations resulting in improved oxygen extraction following exercise.

It is possible that the detected changes in gastrocnemius muscle$\dot{\text{V}}$O_2_ reflect changes in muscle group utilization due to training. Differences in training structure such as inclusion of strength, speed-endurance, or interval training in the program are known to differentially affect muscle function (Burgomaster et al., 1985; Milanović et al., 2015; Vorup et al., 2016). For example, it may be the case that participants replaced other activities (gym sessions, cycling, etc.) with run-training and therefore increased conditioning in the gastrocnemius. Post-exercise muscle$\dot{\text{V}}$O_2_ and the exercise-induced increase in muscle oxygen consumption, Δmus$\dot{\text{V}}$O_2_, were moderately positively correlated with cardio-pulmonary peak$\dot{\text{V}}$O_2_ at the pre-training visit and more weakly at the post-training visit. Consequently, it seems likely that these correlations relate to participant characteristics (e.g., sex, age) rather than to the 6 months of training.

As expected faster marathon completion times were associated with higher cardio-pulmonary peak$\dot{\text{V}}$O_2_, (Joyner and Coyle, 2008) but no convincing relationship was observed between indices of muscle function, post-exercise muscle$\dot{\text{V}}$O_2_ and Δmus$\dot{\text{V}}$O_2_, in gastrocnemius and marathon completion time. This is consistent with cardiopulmonary factors rather than skeletal muscle mitochondrial oxidative capacity (which typically exceeds maximal O_2_ supply) being a dominant factor in peak exercise capacity (Saltin and Calbet, 1985). Previously, cross-sectional data have demonstrated improved metabolic function in athletic vs. inactive subjects (Brizendine et al., 2013). In the study by Brizendine et al. (2013) highly trained endurance athletes, with a mean peak $\dot{\text{V}}$O_2_ = 73.5 ± 9.1 ml/min/kg, were compared to inactive individuals (mean peak $\dot{\text{V}}$O_2_ = 33.7 ± 5.9 ml/min/kg). Other studies also support positive adaptations related to exercise and physical activity (Nagasawa, 2013). However, in studies where arterial occlusions are not performed during the assessment of skeletal muscle function with NIRS, improvements could be attributed to either, or both, improved O_2_ delivery and/or increased mitochondrial capacity.

## Limitations

We did not observe an improvement in peak$\dot{\text{V}}$O_2_ between pre-training and post-training which is contrary to expectation (Joyner and Coyle, 2008). It is possible that detraining following completion of the marathon may have contributed to this; however, the effect of short detraining periods is minimal (Mujika and Padilla, 2000) and is unlikely to fully explain this finding. It is possible that lack of a supervised structured training program, the comparatively moderate intensity of the recommended programme, and/or poor adherence by participants also contributed. Providing structured training programs and support throughout training was beyond the scope of this study. Appropriate methods of collecting information describing participant adherence to training programs (e.g., wearable activity monitors) would be warranted in future similar studies. Nevertheless, our objective was to observe changes that occur in “real-world” subjects undertaking their first marathon; therefore, our findings are likely to be representative of young, healthy adults undertaking endurance events for the first time. Esfarjani et al. randomized participants to structured high intensity training interventions vs. consistent low-intensity running and showed similar, non-significant, increases in peak$\dot{\text{V}}$O_2_ as we report here in the low-intensity group (Esfarjani and Laursen, 2007).

Monitoring training behaviors of the participants in the 6 months prior to the marathon would undoubtedly have provided additional valuable data. However, the objective of this analysis was to investigate if there is an effect of moderate endurance training on skeletal muscle metabolic function in healthy but untrained individuals. The effect of the extent of training was not investigated, although we acknowledge that is likely to have implications for the extent of improvement in skeletal muscle function. All participants included in this analysis completed the marathon and we believe it is fair to assume that this is evidence that, at least minimal, endurance training was undertaken.

Rejection of data due to noise in the NIRS signal was fairly high: ~12% pre-training and ~19% post-training. This could be improved in future studies by using higher cuff inflation pressures to ensure complete arterial occlusions and by preventing movement artifacts through use of a supportive-leg stand to ensure complete muscle relaxation.

## Conclusion

In healthy individuals preparing for their first marathon participation in a 4–6 month training programme is associated with an increase in the ability of gastrocnemius skeletal muscle to utilize oxygen following exercise, implying an improvement in metabolic capacity. Metabolic adaptions can be identified non-invasively using NIRS combined with arterial occlusions within the setting of a supine CPET, although, caution is warranted regarding movement artifacts and insufficient occlusion pressures when using this technique. Post-exercise muscle$\dot{\text{V}}$O_2_ was positively correlated with cardio-pulmonary peak$\dot{\text{V}}$O_2_, particularly at pre-training, but, in terms of performance, cardio-pulmonary peak$\dot{\text{V}}$O_2_ is a better predictor of faster marathon running.

## Author contributions

All authors listed have made a substantial, direct and intellectual contribution to the work, and approved it for publication. SJ: study design, data collection, and post-processing, statistical data analysis, manuscript preparation, and handling of the submission process. AD: study design, data collection, post-processing, and manuscript revisions. AB, GL, CM, and JM: study design, data collection, and manuscript revisions. SS: study design and manuscript revisions. AH: study design, data post-processing, statistical data analysis, and manuscript revisions.

### Conflict of interest statement

The authors declare that the research was conducted in the absence of any commercial or financial relationships that could be construed as a potential conflict of interest.
