# Supplementary material for: Improved Exercise-Related Skeletal Muscle Oxygen Consumption Following Uptake of Endurance Training Measured Using Near-Infrared Spectroscopy
Source: Front Physiol. 2017 Dec 12;8:1018. doi: 10.3389/fphys.2017.01018 (PMC5733097; doi:10.3389/fphys.2017.01018)
Supplement: Supplementary file 1 [file DataSheet1.docx]

# Supplemental information

**Appendix 1. selection process and data flow of NIRS data.**


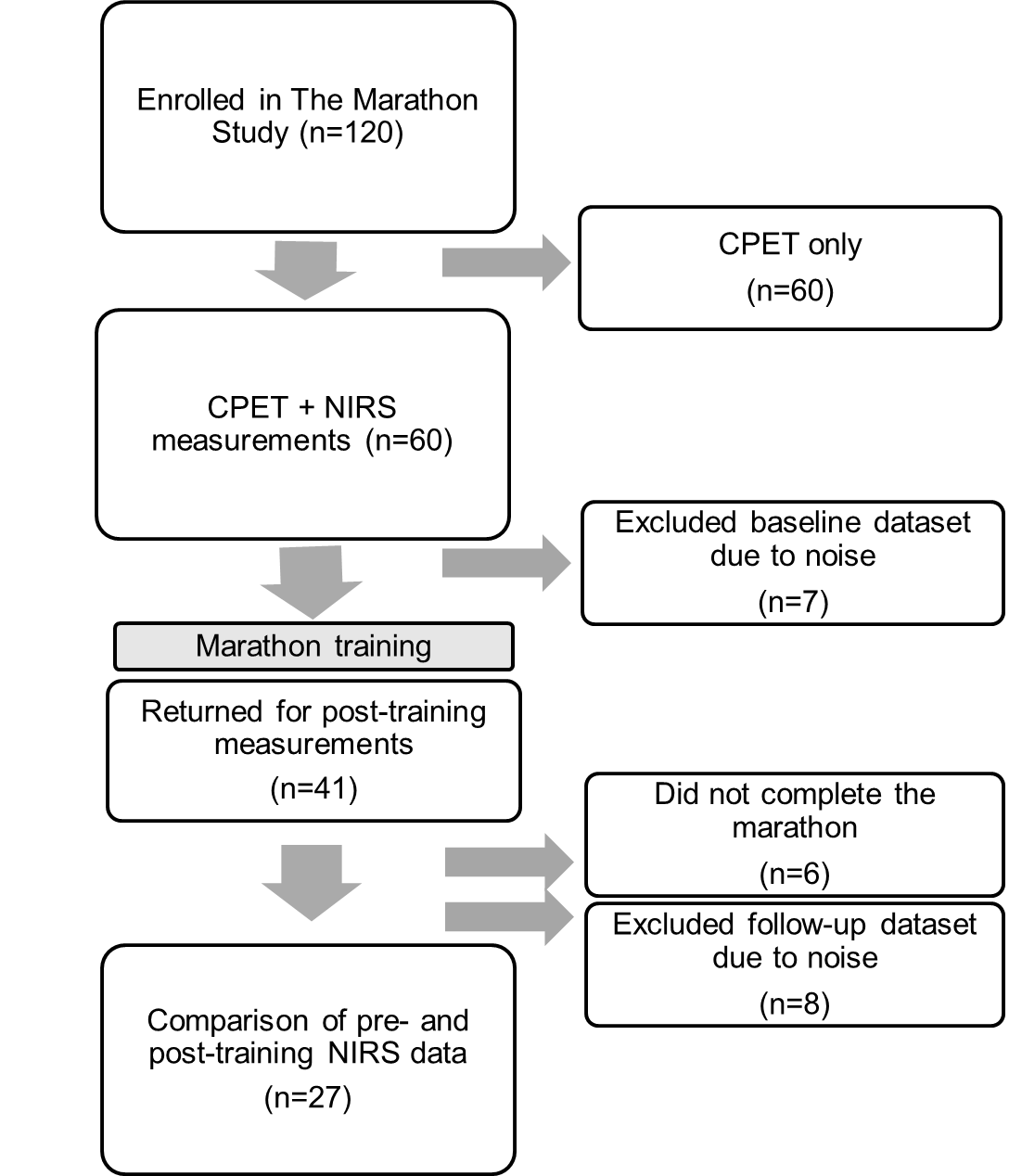


**Appendix 2. Study design highlighting the process of pre-training and post-training measurements. Cardio-pulmonary peakVO_2_ was measured pre- and post-training (via cardio-pulmonary exercise testing (CPET)) and muscle measurements were made at rest and immediately post-exercise at pre- and post-training. VLM; Virgin London Marathon.**

**
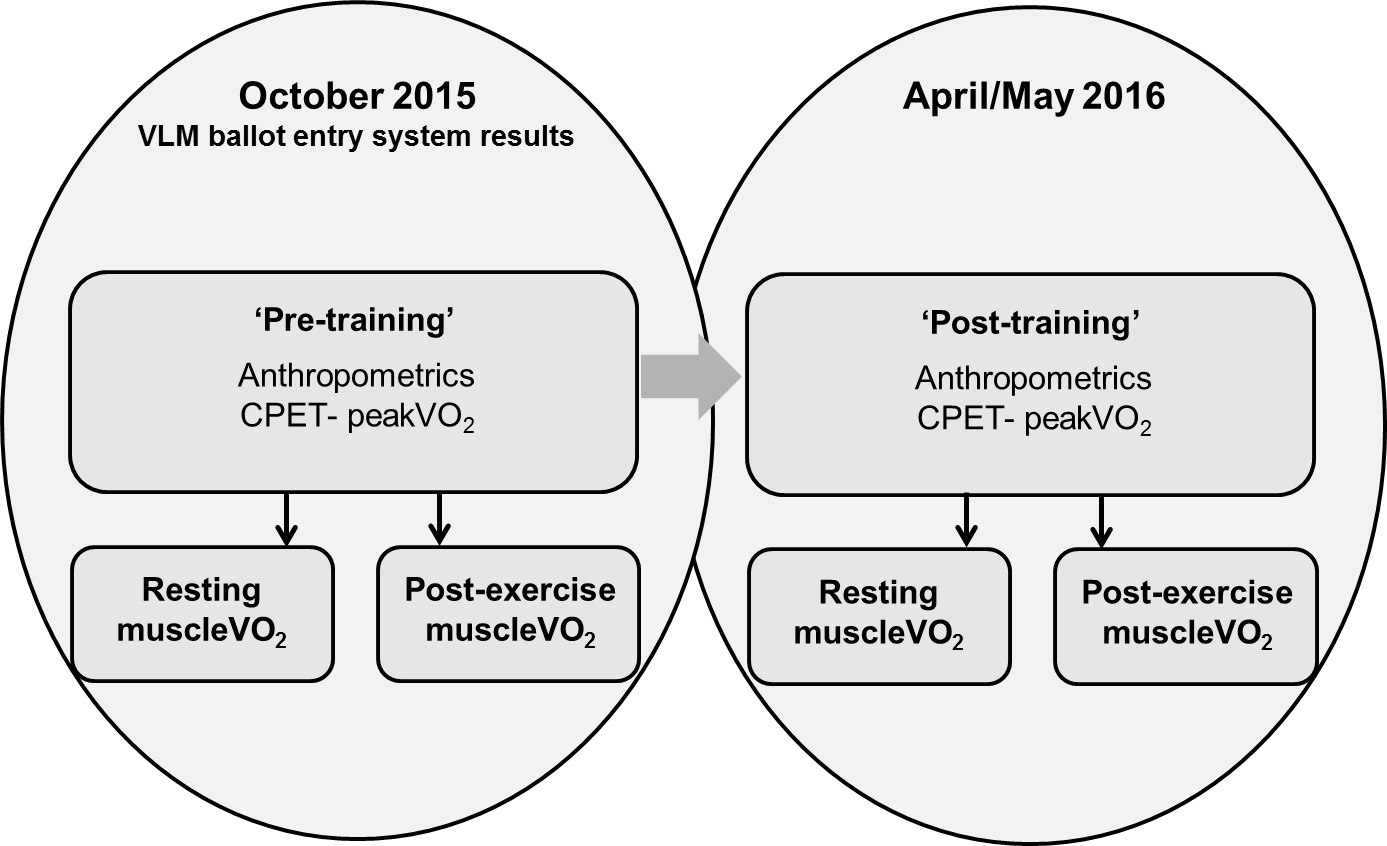
**

**Appendix 3. Results of analysis stratified by gender**

For men and women together higher cardio-pulmonary peakV̇O_2_ values at follow-up were associated with shorter marathon completion times after adjustment for gender and age (r_partial_=-0.58, p= 0.002). In women, higher cardio-pulmonary peakV̇O_2_ values at follow-up were associated with shorter marathon completion times after adjustment for age (r_partial_=-0.68, p= 0.04). In men, this relationship was maintained but was less strong (r_partial_=-0.35, p= 0.19).

For men and women together, there was no convincing relationship between post-exercise muscleV̇O_2_ and marathon completion time (r_partial_=0.31, p=0.14). When stratified by gender the results showed a similar lack of relationship: women (r_partial_=-0.10, p=0.79); men (r_partial_=-0.30, p=0.26).
